# Personality traits of women with hereditary risk for breast/ovarian cancer versus obstetric history and cancer preventive behaviors

**DOI:** 10.1038/s41598-025-87657-6

**Published:** 2025-01-25

**Authors:** Joanna Żurawska, Beata Pięta, Maciej Wilczak, Małgorzata Wojciechowska, Paweł Rzymski, Agnieszka Pieczykolan, Justyna Krysa, Agnieszka Bień

**Affiliations:** 1 Practical Midwifery Science Faculty, Poznan University of Medical Sciences, Poznań, Poland; 2 Department of Maternal and Child Health, Poznan University of Medical Sciences, Poznań, Poland; 3 Chair of Obstetrics Development, Faculty of Health Sciences, Medical University of Lublin, Lublin, Poland

**Keywords:** Personality, BRCA 1 mutation, BRCA 2 mutation, Onco-prophylaxis, Obstetric history, Breast cancer, Human behaviour, Risk factors

## Abstract

The aim of the study is to analyze the relationship between personality traits of women with hereditary predisposition to breast/ovarian cancer and their obstetric history and cancer-preventive behaviors. A total of 357 women, participants of ‘The National Program for Families With Genetic/Familial High Risk for Cancer’, were included in the study. The Neo Five-Factor Inventory (NEO-FFI) and a standardized original questionnaire designed for the purpose of the study were used. Breast ultrasound examination at a younger age was associated with *Extraversion*. *Openness to Experience* was linked with lower number of children, more frequent use of hormonal contraceptives, and younger age at first breast ultrasound examination. Women with higher *Agreeableness* scores were less likely to use contraceptives and underwent their first breast ultrasound later in life. *Conscientiousness* was associated with more frequent use of hormonal contraceptives and younger age at first breast ultrasound examination. Women at increased risk for developing breast/ovarian cancer who used hormonal contraceptives underwent breast ultrasound examinations earlier in life, while those who had breastfed their children chose to have their first mammogram earlier in life. Personality traits affect health-related behaviors and should be taken into account when designing theoretical models as well as interventions regarding health habits.

## Introduction

Breast cancer continues to be a major, global health concern, impacting millions of women annually. It is estimated that approximately one-third of all malignant tumors are attributable to genetic abnormalities. These anomalies are most frequently caused by mutations in the BRCA1 or BRCA2 genes; however, they may also arise from other alterations within the genetic material. Women who have inherited germline mutations in BRCA1 or BRCA2 are at a significantly elevated lifetime risk of developing breast cancer, estimated at 80% and 72%, respectively^1,2^. In Poland, breast palpation, recommended during routine medical visits since the patient age of 29 onwards, remains the core component of preventive care. This test is complemented by recommendations encouraging breast self-examination, advised to all women at the age of 20 and older. Women should undergo breast ultrasound every 24 months between the ages of 20 and 30 and every 12 months since the age of 30 (unless otherwise recommended by their physician). Mammography serves as an important screening tool, routinely performed every 24 months for women aged 45–74 but every 12 months for women aged 45–74 who are at least five years post- breast cancer surgery: both during and after the adjuvant hormone therapy, and after the five-year monitoring period. Suspicious changes detected in mammography require further diagnostic evaluation using methods such as ultrasonography and, in selected cases, magnetic resonance imaging (MRI). Women with significant familial history of breast cancer and carriers of high-risk mutations for breast cancer are included in a care program tailored to their individual risk. Individuals with familial history of breast cancer or other factors indicating a genetic predisposition for breast cancer are offered genetic counseling and genetic testing for carriership of breast cancer mutations, e.g. women with a significant familial history of cancer, personal diagnosis of breast cancer before 40, or a diagnosis of triple-negative breast cancer before 60 years of age^3^. Carriers of one of the three BRCA1 founder mutations are observed in the Polish population, and their cumulative annual risk of developing breast cancer has been estimated at 1.78%^4^. The cumulative incidence of primary breast cancer by the age of 70 in that group of women is 52%. Additionally, following a diagnosis of invasive breast cancer, these women have a high risk of developing a second ipsilateral breast cancer or contralateral breast cancer^5,6^.

Ovarian cancer is a prevalent and aggressive malignancy which presents a significant challenge for the public health. In case of ovarian cancer, preventive strategies are primarily centered on regular gynecological evaluations and an individualized approach to ‘high-risk’ women. In that high-risk population, the use of transvaginal ultrasonography and the measurement of the CA-125 level is recommended, although these modalities are not routinely implemented in the general population^7^. The lack of preventive strategies may contribute to increased morbidity and mortality rates among patients with ovarian cancer diagnosis. Implementing risk prediction models and monitoring for ovarian cancer might prove to be an effective preventive strategy^8^.

According to the research conducted by Kuchenbaecker et al. (2017), the risk of developing breast/ovarian cancer in mutation carriers is modified by both, genetic and non-genetic risk factors. These authors suggest that risk assessment should therefore take into account not only family history, but also other factors which may impact the likelihood of developing cancer^1^. Genetic, environmental, lifestyle factors and their interactions play a crucial role in the development of some of the most common malignancies among women, and recent years have witnessed a growing interest in research about the potential impact of psychological factors, including the ‘Big Five’ personality traits, on cancer susceptibility among females^9,10^.

The ‘Big Five’ personality traits — *Openness to Experience*,* Conscientiousness*,* Extraversion*,* Agreeableness*, and *Neuroticism* — have been studied in various research contexts and the obtained findings suggest a potential association between these traits and cancer prevention strategies among women^11,12^. Individuals with high *Openness to Experience* scores are characterized by curiosity, creativity, willingness to take action, and an inclination towards exploring new ideas. *Conscientiousness* is detected in individuals who are organized, responsible, goal-oriented, and tend to have higher compliance with health recommendations as compared to their peers with lower *Conscientiousness* scores. *Extraversion* is linked with a tendency to display sociable behaviors as well as dominance, and experiencing positive emotions. On the other hand, individuals with high *agreeableness* scores are more inclined towards being kind, gentle, and trustworthy. They are friendly, compliant, compassionate, empathetic towards others, and more willing to offer assistance. Conversely, those with low *Agreeableness* scores tend to be cynical, suspicious, and skeptical. Individuals with high *Neuroticism* scores are typically emotionally unstable, anxious, and moody. Such people are predisposed to experiencing negative emotions and self-pity^12–15^.

Research into the correlation between personality traits in women at risk for developing cancer and their engagement in preventive behaviors represents a relatively new domain of scientific investigation. Understanding these relationships could facilitate the development of personalized cancer-preventive strategies, tailored to the individual personality profiles. Therefore, the aim of this study was to examine the association between personality traits among women genetically predisposed to breast/ovarian cancer, and their obstetric history as well as their cancer-preventive behaviors.

## Materials and methods

### Study design

The study was conducted among patients of the Gynecological-Obstetric Hospital, Poznań University of Medical Sciences (PUMS), Poland, between October 2013 and December 2020. A total of 357 women, participants of ‘The National Program for Families With Genetic/Familial High Risk for Cancer’, were included in the study. The goal of the program is early detection of malignant carcinoma in families with hereditary risk for breast/ovarian cancer. Care over families with high hereditary risk for developing breast/ovarian cancer is offered within the National Oncology Strategy Module I developed by the Ministry of Health, Poland. It involves identification of individuals at high, hereditary genetic predisposition for those malignancies based on genetic testing, personal and familial medical history, and subsequent monitoring of the high-risk groups^7^.

Patients with confirmed BRCA1/2 mutations and a significant family history of the disease (≥ 3 cases among first- or second-degree relatives, including the proband, or first-degree relatives with metachronous or synchronous occurrences of breast and ovarian cancer) are considered to be at the highest-risk for breast cancer. The risk of developing cancer in that group is over 10-fold higher as compared to the general population. Patients without confirmed BRCA1 mutations but with a significant family history (two cases of cancer among first- or second-degree relatives diagnosed before the age of 50) are identified as ‘high-risk’ individuals. The risk of developing cancer in this group is 4-10-fold higher as compared to the general population^14^. The study group, participants of the ‘The National Program for Families With Genetic/Familial High Risk for Cancer’, included 240 healthy women classified as ‘highest’ or ‘high risk’ individuals and 117 women with the diagnosis of cancer. Out of the 117 women, 68% had a history of breast cancer, 3% of both breast and ovarian cancer, and the remaining 29% were diagnosed with ovarian cancer. The study group with the diagnosis of malignancy is considered to be the ‘highest risk’ group of patients^7^.

The following eligibility criteria for genetic testing for breast/ovarian cancer predisposition have been listed: ≥10% probability of pathogenic mutation; breast cancer diagnosis at the age of ≤ 45 years; bilateral breast cancer at ≤ 45 years or ovarian cancer at ≤ 50 years; known predisposing gene mutation for breast and/or ovarian cancer in the family; triple-negative breast cancer diagnosed at ≤ 60 years; non-mucinous epithelial cancer of the ovary or Fallopian tube or primary peritoneal cancer; ancestry linked with the ‘founder effect’, e.g. Ashkenazi Jewish; detection of the somatic BRCA mutations in each type of tumor with the allele frequency of > 30% (if known); at least three breast cancer cases (with at least one diagnosed before the menopause) and/or ovarian cancer in close relatives; at least 2 first-degree relatives with any combination of the following high-risk features: bilateral breast cancer + another malignancy, breast cancer diagnosed < 50 years + prostate cancer or pancreatic cancer < 60 years of age; male breast cancer; breast and ovarian cancer in the same patient; 2 breast cancer cases diagnosed before the age of 50; eligibility for testing for Li-Fraumeni syndrome or Cowden syndrome; pancreatic cancer^16^. Eligibility for the Program constituted the inclusion criterion for the study. Women with familial history of 3 or more breast and/or ovarian cancer cases among first- and second-degree relatives and those with confirmed BRCA1 and BRCA2 or PALB2 gene mutation (regardless of hereditary risk), met the criteria for the high-risk group.

### Data collection

The study used a diagnostic survey technique with two questionnaires: Neo Five-Factor Inventory (NEO-FFI) – Neuroticism Extraversion Openness Five Factor Inventory and a standardized interview questionnaire containing questions designed to characterize the study population.

NEO-FFI by Costa and McCrae (1992; adapted for Polish populations by Zawadzki, Strelau, Szczepaniak, Śliwińska, 2007) includes 60 statements, 12 for each of the scales, which evaluate the following dimensions of the personality: *Neuroticism*, *Extroversion*, *Openness to Experience*, *Agreeableness* and *Conscientiousness.* The statements are self-descriptive, and the participants evaluate their applicability by themselves using a five-point Likert scale, from 1 – ‘I completely disagree’ to 5 – ‘I completely agree’. The score of each of the NEI-FFI scale is summed up to determine the total measure of all personality traits. The raw score of each scale had a possible range of 0–48 points. Higher score was indicative of a higher prevalence of a given personality trait. The α Cronbach coefficient for internal consistency was: 0.86 for *Neuroticism*, 0.77 for *Extroversion*, 0.73 for *Openness to Experience*, 0.68 for *Agreeableness*, and 0.81 for *Conscientiousness*^11^.

The standardized questionnaire included questions concerning obstetric history, i.e. miscarriage, number of pregnancies, postpartum breastfeeding, use of contraception, and engagement in cancer-preventive behaviors such as breast self-examination, breast ultrasound and mammograms.

The study was performed in accordance with the Helsinki Declaration and approved by the PUMS Bioethics Committee (approval no. 706/16). The respondents were informed that participation was voluntary, and that study results were anonymous and to be used exclusively for research purposes. Informed consent was obtained all subjects.

### Statistical analysis

Statistical analysis was conducted using StatSoft Statistica (v. 13.3). Quantitative data were presented using means (M), standard deviations (SD), and median (Me), minimum (Min), maximum (Max) values; qualitative data were presented using numbers and percentages. The Shapiro-Wilk test was used to investigate the distribution of the quantitative data. One-way analysis of variance ANOVA (F) was used to test the dependence of one or more variables (equal variances). Linear regression analysis was used to test the mediator model. A series of regression analyses using the enter method was also conducted, which involves incorporating all explanatory variables into the model. Result interpretation in this method involves comparing the values of the β coefficient, which is interpreted in terms of the direction and strength dependence of all predictors. The adjusted- square is used to interpret the fit of the regression model. The p-value of < 0.05 was considered as statistically significant.

## Results

Mean age of the study population was 49.73 years (SD = 11.01). The majority of the women were city dwellers (76.19%), with secondary education (39.49%), and no history of miscarriage (81.51%). The highest number of women were secundiparas (43.14%), with history of breastfeeding (80.11%). Most respondents reported no hormonal contraceptive use (74.51%), and regular breast self-exam (84.31%). Disease prevention was the main reason for prophylactic screening in the vast majority of cases (80.67%). Mean age at first breast ultrasound was 40.39 (SD = 7.44) years and at first mammogram was 45.02 (SD = 6.22) years (Table 1).

**Table 1:** Characteristics of the study population (*n* = 357).

Characteristics of the study population	*N*	%
Age		
M (SD); Me	49.73 (11.01); 38.00 26.00–79.00	
Min–Max		
Area of residence		
Urban	272	76.19
Rural	85	23.81
Education		
Primary/vocational	82	22.96
Secondary	141	39.49
Higher	134	37.54
Miscarriage		
Yes	66	18.49
No	291	81.51
Number of pregnancies		
1	74	20.73
2	154	43.14
≥ 3	107	29.97
0	22	6.16
Breastfeeding		
Yes	286	80.11
No	71	19.89
Hormonal contraceptives		
Yes	91	25.49
No	266	74.51
Regular breast self-exam		
Yes	301	84.31
No	56	15.69
Reason for screening		
Prophylaxis (*participation in screening tests as part of national preventive programs*)	288	80.67
Lesion	126	35.29
Mastalgia	15	4.20
Age at first breast ultrasound		
M (SD); Me	40.39 (7.44); 40.00 19.00–65.00	
Min–Max		
Age at first mammogram		
M (SD); Me	45.02 (6.22); 45.00	
Min–Max	31.00–65.00	

*M* mean, *SD* standard deviation, *Me* median.

Mean values for the investigated personality traits are presented in Table 2. *Neuroticism* (M = 21.72; SD = 7.73) was the least frequent, while *Conscientiousness* (M = 35.06; SD = 5.19) and *Agreeableness* (M = 31.78; SD = 4.94) were the most frequent personality traits.

**Table 2:** Prevalence of the personality traits in the study population.

NEO-FII	M	SD	Me	Min.	Max.
Neuroticism	21.72	7.73	21.00	2.00	47.00
Extraversion	28.75	5.81	29.00	12.00	46.00
Openness to experience	25.47	5.13	25.00	12.00	41.00
Agreeableness	31.78	4.94	32.00	10.00	45.00
Conscientiousness	35.06	5.19	35.00	20.00	48.00

*M* mean, *SD* standard deviation, *Me* median.

Regression analysis for the variables of cancer-preventive behaviors and obstetric history is presented in Table 3. *Openness to Experience* (*β = -0.209; p = 0.000*) was a statistically significant predictor for the number of pregnancies, with higher *Openness to Experience* score corresponding to lower number of pregnancies. *Openness to Experience* (*β = 0.149; p = 0.006*), *Agreeableness* (*β = -0.149; p = 0.009*) and *Conscientiousness* (*β = 0.183; p = 0.001*) were statistically significant predictors for the use of contraceptives. Women with high scores for *Openness to Experience* and *Conscientiousness* used contraceptives more frequently, whereas those with high *Agreeableness* scores did not use contraceptives. *Extraversion* (*β = -0.141; p = 0.014*), *Openness to Experience* (*β = -0.178; p = 0.001*), *Agreeableness* (*β = 0.114; p = 0.042*) and *Conscientiousness* (*β = -0.181; p = 0.001*) were statistically significant predictors for age at first breast ultrasound. Women with high scores for *Extraversion*, *Openness to Experience* and *Conscientiousness* as well as those with low *Agreeablenes*s scores underwent their first breast ultrasound at a younger age. No statistically significant predictors were found for the following variables: history of breastfeeding, breast self-exam, and age at first mammogram (*p* < 0.05).

**Table 3:** Univariate regression analysis of cancer-preventive behaviors and obstetric history versus the big five personality traits (NEO-FFI).

Variable	Number of pregnancies F = 3.713; *p* < 0.01; *R* = 0.224; R^2^ = 0.050; adjusted-R^2^ = 0.037					Hormonal contraception F = 4.521; *p* < 0.001; *R* = 0.246; R^2^ = 0.061; adjusted-R^2^ = 0.047					Age at first breast ultrasound F = 6.998; *p* < 0.001; *R* = 0.301; R^2^ = 0.091; adjusted-R^2^ = 0.078				
	B	SE	β	t	*p*	B	SE	β	t	*p*	B	SE	β	t	*p*
(Constant)	2.691	0.772		3.486	0.001	0.059	0.293		0.201	0.841	57.577	4.740		12.148	0.000
Neuroticism	0.003	0.009	0.024	0.384	0.701	− 0.004	0.003	− 0.071	− 1.162	0.246	− 0.104	0.055	− 0.112	− 1.876	0.061
Extraversion	0.006	0.012	0.031	0.537	0.592	− 0.006	0.004	− 0.074	− 1.277	0.202	− 0.173	0.070	− 0.141	− 2.463	**0.014**
Openness to experience	− 0.047	0.012	− 0.209	− 3.874	**0.000**	0.013	0.005	0.149	2.784	**0.006**	− 0.249	0.074	− 0.178	− 3.364	**0.001**
Agreeableness	0.025	0.013	0.109	1.909	0.057	− 0.013	0.005	− 0.149	− 2.636	**0.009**	0.164	0.080	0.114	2.041	**0.042**
Conscientiousness	− 0.013	0.012	− 0.058	− 1.027	0.305	0.015	0.005	0.183	3.242	**0.001**	− 0.250	0.077	− 0.181	− 3.254	**0.001**

Significant values are in bold.

Results of the regression analysis for cancer-preventive behaviors are presented in Table 4. Number of pregnancies (*β = 3.774; p = 0.000*) and use of contraceptives (*β = -0.227; p = 0.000*) proved to be statistically significant predictors for age at first breast ultrasound. Women with lower number of pregnancies and those using contraceptives underwent their first breast ultrasound at a younger age as compared to their peers with higher number of pregnancies scores and contraceptive non-users. Number of pregnancies (*β = 2.305; p = 0.022*) and history of breastfeeding (*β = -0.168; p = 0.009*) were statistically significant predictors for age at first mammogram. Women with lower number of pregnancies and history of breastfeeding underwent their first mammogram at a younger age as compared to women with higher number of pregnancies and no history of breastfeeding. No statistically significant predictors were found for the breast self-exam variable (*p* > 0.05).

**Table 4:** Univariate regression analysis of cancer-preventive behaviors and obstetric history.

Predictor	Age at first breast ultrasound F = 15.746; *p* < 0.001; *R* = 0.390; R^2^ = 0.152 Adjusted-*R*^2^ = 0.142					Age at first mammogram F = 3.370; *p* < 0.01; *R* = 0.220; R^2^ = 0.048 adjusted-*R*^2^ = 0.034				
	B	SE	β	t	*p*	B	SE	β	t	*p*
(Constant)	39.196	0.951		41.224	0.000	45.904	1.070		42.920	0.000
Miscarriage	− 1.902	1.015	− 0.104	− 1.875	0.062	− 1.664	1.046	− 0.108	− 1.591	0.113
Number of pregnancies	1.401	0.371	0.225	3.774	**0.000**	0.892	0.387	0.165	2.305	**0.022**
Breastfeeding	− 0.050	0.938	− 0.003	− 0.054	0.957	− 2.718	1.067	− 0.161	− 2.547	**0.011**
Contraceptives	− 4.926	0.816	− 0.302	− 6.037	**0.000**	− 1.615	1.012	− 0.097	− 1.596	0.112

Significant values are in bold.

## Discussion

The incidence of breast cancer continues to increase globally. Consequently, it is imperative to evaluate the role of modifiable risk factors to better guide the development of future primary prevention strategies^17,18^. In light of the fact that the development of breast cancer is affected by a combination of biological, psychological, and environmental determinants, the causal mechanism cannot be directly attributed to a single risk factor^1,19^.

Although numerous studies focus on the genetic and the environmental factors, recent years have witnessed a growing interest in the impact of the psychological factors, particularly the ‘Big Five’ personality traits, on cancer-preventive behaviors. Understanding of these correlations might pave the way for personalized cancer-preventive strategies, which would be based on the personality profile of a given individual. Our study allowed to demonstrate an association between the personality traits of women with a hereditary predisposition to breast/ovarian cancer and their obstetric history as well as their cancer-preventive behaviors. Confirmed carriership of genetic mutations associated with breast/ovarian cancer necessitates that these women be under the care of a genetic counselor and implement appropriate preventive measures, including the following: breast self-examinations since the age of 18; breast imaging tests (until 25 - ultrasound and MRI, since the age of 35 - mammogram) very six months; earlier rather than later-in-life motherhood; breastfeeding for as long as possible; gynecological examinations every six months since the age of 30; annual transvaginal ovarian ultrasound test; and possible prophylactic removal of the ovaries and Fallopian tubes after completing their childbearing potential, ideally between ages 35 and 40 (or slightly later for patients with BRCA2)^20–22^.

Our analysis of the relationship between *Openness to Experience* and childbearing decisions revealed that women with higher scores for this personality trait were less likely to conceive as compared to those with lower scores for *Openness to Experience*^12^. Their adventurous and daring nature may lead them to prioritize personal development, career advancement, and other life experiences before choosing to start a family. When they do decide to have children, they may opt for fewer offspring. Obviously, it is important to recognize that the relationship between personality traits and reproductive plans is more complex and multifactorial. Individual life goals, cultural norms, and socio-economic status can also influence reproductive decisions. Furthermore, the Big Five personality traits do not operate in isolation but interact both, with one another and with other external factors, shaping specific behaviors and choices^23,24^. Moreover, Friebel et al. (2014), revealed a statistically significant reduction in breast cancer risk among women who gave birth and were carriers of the BRCA1 and BRCA2 gene mutations. A meta-analysis of cohort studies demonstrated a statistically significantly reduced risk for women with 3 + live births as compared to nulliparas and with 4 + live births as compared to nulliparas, with each subsequent birth as an additional risk-reduction factor^25^. Advanced maternal age at first delivery is also linked with elevated risk for developing breast cancer. Higher disease prevalence observed among younger (< 45 years) women correlates with lower parity rates within that age group. Given the current trends in delayed childbearing, the incidence of breast cancer will most likely continue to rise^26^.

Lower parity is an additional, well-established risk factor for developing breast cancer. According to Lefrère et al., breast cancer risk is reduced by approximately 7% for each full-term pregnancy, while it increases by about 5% for each additional year that age of the first pregnancy has been delayed^27^. As far as the impact of parity on ovarian cancer risk is concerned, a significant reduction in risk is observed among multiparous (4+) women as compared to nulliparas^25^.

Women with a hereditary predisposition to breast cancer often face unique challenges in managing their health. Genetic factors that significantly increase the likelihood of developing breast cancer prompt many women to adopt preventive strategies, including regular screening tests and lifestyle modifications^25^. The choice of a contraceptive method may be influenced by socio-economic and demographic factors, as well as personal preferences. Tolerance of a contraceptive method, its impact on cognitive functions, emotions, and the overall well-being may be determined by personality traits. Decisions regarding the use or non-use of oral contraceptives may stem from neurophysiological differences among women. Additionally, personality traits may affect how women manage physical and/or psychological discomfort associated with hormonal fluctuations during the menstrual cycle. Women with higher *Neuroticism* scores, for example, may opt for oral contraception to avoid the inconveniences related to their menstrual cycle^28^. In our study, the analysis of hormonal contraceptive use among the study participants revealed that individuals with high scores in *Openness to Experience* and *Conscientiousness* were more likely to use hormonal contraception. According to Pletzer et al., women exhibiting higher levels of *Agreeableness* and *Extraversion* more frequently chose intrauterine devices (IUDs) as their contraceptive method when compared to women with other personality traits, who were more inclined to select oral contraceptives. These findings suggest that personality is more strongly associated with the choice of contraceptive method type than with the decision between hormonal and non-hormonal methods of contraception^28^. Conversely, Beltz et al., found no significant differences in personality traits between individuals who did and those who did not use oral contraceptives^29^.

Individuals with high *Conscientiousness* scores possess decision-making capabilities and consider the long-term consequences of their choices^30^. When selecting a method of contraception, individuals with higher *Agreeableness* scores are more likely to consider factors such as effectiveness, side effects, compatibility with their lifestyle, and their overall health status. This tailored approach, aligned with their needs and preferences, enhances the likelihood of effective contraceptive use. Their attention to detail and commitment to following instructions further reduces the probability of contraceptive failure^30^. According to the literature, evidence regarding the potential association between the use of hormonal contraceptives and the risk of developing breast cancer remains inconclusive, particularly with regard to methods that contain only progestin^31–33^. A meta-analysis of case-control studies by Friebel et al. (2014), suggested that the use of oral contraceptives did not influence breast cancer prevalence among women with genetic and hereditary predispositions, as compared to those who never used oral contraception. However, the combined hazard ratios from the cohort studies demonstrated an elevated risk of developing breast cancer among the users of oral contraceptives^25^. In turn, Cohen et al. (2023), demonstrated that prolonged use of hormonal contraception increased the risk of breast cancer among women with inherited predisposition^34^. In the case of ovarian cancer, numerous studies and systematic reviews have consistently reported a reduced risk of ovarian cancer associated with the use of hormonal contraceptives among women from average and high-risk groups^35,36^. The use of oral contraceptives for over 1 year has been found to significantly reduce the risk of ovarian cancer among BRCA1 mutation carriers (from 33 to 80%). Furthermore, women who have used oral contraceptives for 10 + years experience a decrease in ovarian cancer incidence by over 50%^25,36,37^.

Other studies have also reported a significantly reduced risk of developing ovarian cancer among BRCA1 and BRCA2 mutation carriers who had used combined oral contraceptives. The same research also demonstrated a decreased risk of ovarian cancer with prolonged use of this form of contraception^36,38,39^.

Our research also indicated that women with higher scores of *Agreeablenes*s are less likely to use hormonal contraception as compared to those with lower levels of this trait. *Agreeableness* encompasses characteristics such as compassion, willingness to cooperate, and a desire to avoid conflict^30^. Women with higher levels of *Agreeableness* may prioritize concerns about the potential side effects and health risks associated with hormonal contraception over contraceptive benefits. This heightened sensitivity to possible negative health impacts might lead them to seek alternative contraceptive methods, perceived as safer or less invasive. Additionally, individuals with hereditary predisposition to breast/ovarian cancers may have heightened health awareness, but may also experience increased anxiety or fear regarding the potential development of these diseases. As a result, they may approach contraceptive decision-making with greater caution and deliberation^12^.

Understanding how personality traits influence the decisions regarding contraceptive use should be taken into consideration by the healthcare providers who will support women with a hereditary predisposition to breast/ovarian cancers in making informed contraceptive choices. It is also important to emphasize the significance of personalized, patient-centered care which takes into account individual differences in risk perception, personality traits, and familial medical history^21^. On the other hand, it is important to recognize that personality traits operate within a broader context influenced by social, cultural, and environmental factors. While individuals with higher *Openness to Experience* and *Conscientiousness* scores may engage in responsible contraceptive behaviors, barriers such as limited access to healthcare services, the stigma surrounding contraception, or misinformation can still hinder the effective use of contraceptives^40^.

As far as cancer prevention is concerned, early detection is often the key to effective treatment and favorable outcomes. In case of breast cancer, women with a hereditary predisposition are at an elevated risk for developing the malignancy, which necessitates proactive measures, including breast self-examination and early screening^5^. The most common imaging modalities for breast cancer include ultrasonography, mammography, and magnetic resonance imaging^1^. Breast ultrasonography is an essential tool in early detection of breast cancer, providing a non-invasive method of evaluating breast tissue and identifying abnormalities. In case of individuals with hereditary predisposition to breast cancer, it is recommended that breast ultrasound screenings begin at the age of 25, as early screening increases the likelihood of detecting abnormalities at an earlier stage, thereby improving treatment efficacy and prognosis^20,41^.

In our study, individuals with higher scores of *Extraversion*,* Openness to Experience*, and *Conscientiousness* tended to undergo their first breast ultrasound at a younger age. *Extraversion*, in particular, may be associated with a stronger inclination towards control, mitigating potential threats, or compliance with medical recommendations. It is consistent with the findings of Bahat et al. (2021), who demonstrated that health-promoting behaviors, including compliance with preventive screening recommendations, are influenced by the strength and intensity of a given personality trait. Individuals with high *Extraversion* scores are more likely to seek detailed information about their health status and demonstrate a greater need for support from healthcare professionals. Moreover, extraverted individuals are more inclined to adhere to medical advice regarding health-promoting behaviors, such as breast self-examination, mammography, and clinical breast exams^15^. Women with high *Openness to Experience* scores are more likely to initiate health-promoting and preventive behaviors in accordance with medical recommendations, driven by their curiosity and information-seeking behavior^10,15^. Also, *Openness to Experience* was proven to be a significant predictor of breast lump check and mammogram in women aged 65+^42^. Conscientious individuals are known for their organization, diligence, and goal-oriented behavior. These traits may contribute to a meticulous approach to health-related decisions, prioritizing cancer screenings and adhering to medical recommendations. As highlighted by Friedman et al., such individuals often prioritize preventive health measures^43,44^. In turn, Bahat et al., demonstrated that higher levels of conscientiousness are associated with adherence to recommendations for undergoing ultrasound examinations. Furthermore, higher *Extraversion*, as well as *Conscientiousness*, and *Openness to Experience* positively correlated with the frequency of self-examinations, regardless of the age group, ethnic background, or educational level of the respondents^15^.

Although advances in screening and early detection have notably improved the patient outcomes, some women with the trait of *Agreeableness* may delay undergoing breast ultrasound examinations. Our research demonstrated that women with a hereditary predisposition to breast/ovarian cancers who exhibit lower levels of *Agreeableness* tend to undergo breast ultrasound screenings at a younger age compared to women with higher levels of *Agreeableness*. One possible explanation is that less agreeable women are more assertive and proactive in managing their own health^12^. They may possess an increased sense of self-awareness and autonomy, which encourages them to take control of health-related decisions at an earlier stage in life^15^. While personality traits can positively impact cancer-preventive behaviors, individual factors such as familial risk factors and cultural beliefs also play a role in determining patient age at first breast ultrasound screening. Additionally, access to healthcare services as well as socioeconomic factors can affect screening-related behaviors, highlighting the need to ensure equitable access to preventive care for all individuals^45^.

Our findings indicated that women with lower number of pregnancies and with history of breastfeeding underwent their first mammogram at a younger age as compared to women with higher number of pregnancies and no history of breastfeeding. A study conducted among Iranian women by Moghaddam Tabrizi et al. (2018), investigating the determinants of breast cancer screening through mammography, found no significant associations with the number of children, duration of breastfeeding, or history of breastfeeding^46^. In turn, maternal age at conception was not a predictor for mammography screening among Iranian women^47^. Pokora et al. (2022), found no link between breastfeeding and the participation rates in screening for breast cancer^48^. The study conducted by Moreira CB et al. (2018), among Brazilian women, indicates that adherence to mammography screening is more common among women who are married or in a stable relationship and have one or two children^49^. Supporting this finding, the researchers who investigated the predictive factors of perceived barriers to mammography among Korean women found that the number of children and marital status significantly affected adherence to mammography screening. The perceived likelihood of undergoing this test was higher among married women with one to three children^50^.

The literature offers data which suggest that breastfeeding provides a protective effect against breast cancer. The risk reduction is more pronounced with prolonged breastfeeding. Specifically, breastfeeding for over 12 months is associated with a 26% decrease in the risk of developing breast cancer^51,52^. Our research indicates that women who had breastfed their children exhibited greater awareness of secondary prevention measures. They tend to undergo their first mammogram at a younger age as compared to women who had not breastfed. Cross-sectional studies conducted by Lera et al. (2020), among Ethiopian women aged 20–65, demonstrated that women who breastfed their children for 13–24 months were more likely to perform breast self-examinations as compared to women with shorter duration of breastfeeding^53^. Research conducted by Carey and El-Zaemey (2020), in Western Australia, demonstrated that participation in breast mammography screening was affected by lifestyle and occupational factors. The likelihood of undergoing mammography was higher among women with history of HRT and those working in clerical occupations or home duties as compared to those in professional or technical occupations^54^.

The analysis of our findings regarding the association between obstetric history and cancer-preventive behaviors revealed that women using contraceptives underwent their first breast ultrasound at a younger age as compared to those not using contraception. Similarly, Irmov et al. (2017), also reported that contraceptive use effected the frequency of preventive examinations in women. Women who use hormonal contraception report a higher frequency of regular preventive gynecologic examinations^55^. A cross-sectional study by Manisha BK et al. (2023), conducted among Nepali women, demonstrated that the use of contraceptives is a significant factor affecting the practice of breast self-examination^56^. Mignot et al. (2019), analyzed data from a cohort study of women in France and found that patients seeking contraceptive care from physicians more often underwent cytological examinations. Women who did not use contraception or used non-prescription contraception underwent cytological screenings significantly less regularly as compared to other women^40^.

## Summary

Personality traits are a complex and multifactorial issue, with numerous – other than personality alone – variables affecting decisions related to cancer prevention strategies. Still, understanding the relationship between personality traits and healthcare decisions can provide valuable insights for healthcare providers. Knowledge about the role of personality in shaping prevention behaviors allows physicians to offer a more individualized approach and to meet the needs of their patients, ultimately leading to more personalized and effective health interventions. It is also essential for patients to be aware of their personality traits and use them to make informed lifestyle choices which affect their health^44^. The study of the relationship between personality traits in women with hereditary risk for breast/ovarian cancer, their obstetric history, and preventive behaviors demonstrated a correlation between personality traits (e.g., openness to experience, conscientiousness) and the use of contraception, parity, and age at first breast ultrasound. Additionally, parity and breastfeeding were found to be linked with the age at first mammogram. The results suggest that incorporating personality traits may contribute to the development of personalized cancer prevention strategies. Our findings also indicate that personality may serve as a pivotal mediator of health-related behaviors and should be incorporated into future theoretical models and interventions aimed at promoting a healthy lifestyle. As the complex relationship between personality and health continues to be investigated, integrating these insights into public health strategies and individualized healthcare plans offers hope to have a healthier society^57,58^.

### Strengths and limitations of the study

Awareness of the risks, as well as diagnostic and therapeutic options, may significantly affect the decisions related to family planning and risk management among younger individuals from families with a genetic predisposition to breast/ovarian cancer (higher attendance rates at early screening and diagnostic procedures). Inclusion of the psychological/personality profiles might facilitate communication and provide tailored support for individuals requiring continuous assistance in cancer-preventive measures.

This study allows to identify the psychological factors and those personality traits which play a significant role when assessing the risk for developing breast cancer. As the literature offers a limited number of sources regarding this matter, this research is innovative and contributes to expanding our knowledge about the matter. Consequently, it may contribute to establishing more personalized primary and secondary prevention strategies. This study is not without limitations, chief among them the restricted scope of the research, which was confined to a single province in Poland. Additionally, the study did not analyze the relationship between the ‘Big Five’ personality traits and other health-promoting behaviors such as engagement in physical activity or dietary habits. Responses to questions regarding past behaviors may be subject to recall bias, while questions concerning personality traits may be influenced by self-report bias.

## Conclusions

Women with an elevated risk of developing breast/ovarian cancer and with higher *Extraversion* scores tend to undergo their first breast ultrasonography at a younger age. Those with higher *Openness to Experience* scores are less likely to have children, use hormonal contraceptives more frequently, and have their first breast ultrasonography at a younger age. Women with higher *Agreeableness* scores are less likely to use contraceptives and tend to undergo their first breast ultrasound at an older age. Individuals with higher *Conscientiousness* scores are more likely to use hormonal contraception and to have their first breast ultrasonography at a younger age. Contraceptive use and lower number of pregnancies among women with an elevated risk of developing breast/ovarian cancer are associated with younger age at first breast ultrasonography. Conversely, lower number of pregnancies and breastfeeding are correlated with younger age at first mammogram.

## Data Availability

The datasets used and analysed during the current study available from the corresponding author on reasonable request.
